# Patterns of health-related quality of life and patterns associated with health risks among Rhode Island adults

**DOI:** 10.1186/1477-7525-6-49

**Published:** 2008-07-11

**Authors:** Yongwen Jiang, Jana Earl Hesser

**Affiliations:** 1 Center for Health Data and Analysis, Rhode Island Department of Health, Providence, Rhode Island, USA

## Abstract

**Background:**

Health-related quality of life (HRQOL) has become an important consideration in assessing the impact of chronic disease on individuals as well as in populations. HRQOL is often assessed using multiple indicators. The authors sought to determine if multiple indicators of HRQOL could be used to characterize patterns of HRQOL in a population, and if so, to examine the association between such patterns and demographic, health risk and health condition covariates.

**Methods:**

Data from Rhode Island's 2004 Behavioral Risk Factor Surveillance System (BRFSS) were used for this analysis. The BRFSS is a population-based random-digit-dialed telephone survey of adults ages 18 and older. In 2004 RI's BRFSS interviewed 3,999 respondents. A latent class regression (LCR) model, using 9 BRFSS HRQOL indicators, was used to determine latent classes of HRQOL for RI adults and to model the relationship between latent class membership and covariates.

**Results:**

RI adults were categorized into four latent classes of HRQOL. Class 1 (76%) was characterized by good physical and mental HRQOL; Class 2 (9%) was characterized as having physically related poor HRQOL; Class 3 (11%) was characterized as having mentally related poor HRQOL; and Class 4 (4%) as having both physically and mentally related poor HRQOL. Class 2 was associated with older age, being female, unable to work, disabled, or unemployed, no participation in leisure time physical activity, or with having asthma or diabetes. Class 3 was associated with being female, current smoking, or having asthma or disability. Class 4 was associated with almost all the same predictors of Classes 2 and 3, i.e. older age, being female, unable to work, disabled, or unemployed, no participation in leisure time physical activity, current smoking, with having asthma or diabetes, or with low income.

**Conclusion:**

Using a LCR model, the authors found 4 distinct patterns of HRQOL among RI adults. The largest class was associated with good HRQOL; three smaller classes were associated with poor HRQOL. We identified the characteristics of subgroups at higher-risk for each of the three classes of poor HRQOL. Focusing interventions on the high-risk populations may be one approach to improving HRQOL in RI.

## Background

Two overarching US Healthy People 2010 objectives are "to increase quality and years of healthy life," and "to eliminate health disparities" [1]. With the transition from infectious disease and acute illness to chronic disease and degenerative illness as leading causes of death, quality of life has become an important aspect in assessing the burden of disease.

Health-related quality of life (HRQOL) refers to an individual's perception of their own physical and mental health, and their ability to react to factors in the physical and social environments [1]. It also includes aspects of life that affect perceived physical or mental health [2-4]. HRQOL is predictive of morbidity and mortality and is recognized as an important public health indicator [2-4]. It is increasingly used to monitor the burden of disease in a population [3] and is taken into consideration in decision-making regarding resource allocation, intervention design, and chronic disease management [5]. Continuous monitoring of population HRQOL gives public health agencies data they need to assess, protect, and promote population health. Tracking population HRQOL helps identify health disparities, evaluate progress on achieving broad health goals such as Healthy People 2010, and informs public health policy [4].

HRQOL is subjective and, therefore, cannot be measured directly [1]. Individual HRQOL indicators have been developed to assess different aspects of HRQOL. Building on our earlier analyses of HRQOL indicators for the Rhode Island population [6], we wished to answer the following questions: Is it possible to characterize HRQOL with summary measures so health planners can track Rhode Island's HRQOL over time? Is it possible to characterize patterns of HRQOL in Rhode Island's population? What are the predictors of different patterns of HRQOL? Can we quantify the percentage of RI's population that has good versus compromised HRQOL? To explore these questions, we applied a latent class regression model (LCR) to data from RI's 2004 BRFSS, including 9 HRQOL indicators.

## Methods

A LCR model is a statistical model for categorical data that can be used to identify classes of respondents and examine the association between covariates and latent class membership [7]. In this study, a LCR model was fit to identify a pattern of HRQOL in the Rhode Island population, to determine what proportion of the population can be characterized by classes of HRQOL within this pattern, and to examine associations between demographics, health risks, and health conditions and classes of HRQOL among Rhode Island adults, adjusted for all other variables in the model. We used the nine HRQOL indicators from the 2004 Rhode Island BRFSS data.

The BRFSS is an ongoing, state-based, cross-sectional, annual random-digit-dialed telephone survey of the non-institutionalized civilian population ages 18 years or older. It has been sponsored since 1984 by the Centers for Disease Control and Prevention (CDC), which provides funding, methodological specifications, and technical assistance to participating states. The BRFSS is conducted currently in all 50 states, the District of Columbia, Guam, Puerto Rico, and the Virgin Islands [8]. The survey monitors the prevalence of key health- and safety-related behaviors and characteristics for the leading causes of disease and death among adults [8,9]. In 1993, the CDC developed the "Healthy Days Measures", a set of 4 questions for the Behavioral Risk Factor Surveillance System (BRFSS) core survey [5]. CDC designed these questions to measure HRQOL in the general population and to assess an individual's perceptions of their general health status, physical and mental health, and activity limitations related to physical or mental health [5]. In response to growing interest in HRQOL, CDC developed an expanded set of questions, which have been available for use with the BRFSS since 1995. These questions measure multiple dimensions of HRQOL including "specific types of activity limitation and common physical and emotional symptoms" [10]. Rhode Island has included the "core" and expanded HRQOL questions on its annual BRFSS from 1997 through 2006.

### Data source

The authors used Rhode Island's 2004 BRFSS data for this analysis. A professional survey organization under contract to the Rhode Island Department of Health conducted Rhode Island's 2004 BRFSS. From January through December 2004, the Rhode Island BRFSS conducted approximately 333 random-digit-dialed telephone interviews each month with adults ages 18 and older, for a total of 3,999 (1,531 males and 2,468 females) during the calendar year. The response rate in 2004, as defined by the Council of American Survey Research Organizations (CASRO), was 51%. Rhode Island's 2004 BRFSS data and technical details are available upon request from the Center for Health Data and Analysis, Rhode Island Department of Health [11].

### Indicators

This study used data from nine HRQOL questions. The first question asked respondents to rate their general health as excellent, very good, good, fair, or poor. These responses were dichotomized into (1) excellent, very good, or good and (2) fair or poor. The remaining eight questions asked respondents to estimate the frequency of various conditions during the previous 30 days as follows: "How many days did poor physical or mental health keep you from doing your usual activities?" (Activity limitation); "How many days was your physical health, which includes physical illness or injury, not good?" (Physically unhealthy); "How many days did pain make it difficult to do your usual activities?" (Pain related activity limitation); "How many days have you felt very healthy and full of energy?" (We used the converse for Lack of energy.); "How many days did you feel you did not get enough rest or sleep?" (Lack of rest/sleep), "How many days did you feel worried, tense, or anxious?" (Worried/tense/anxious); "How many days was your mental health, which includes stress, depression, and problems with emotions, not good?" (Mentally unhealthy); and "How many days did you feel sad, blue, or depressed?" (Sad/blue/depressed) [6,8,12,13]. Responses were dichotomized into 0 to 13 (infrequent) and 14 to 30 (frequent) unhealthy days [14,13]. The authors used the cut-off of 14 or more days vs. 13 or fewer days because most of the publications we reviewed utilized this convention in analyzing the BRFSS HRQOL indicators [2,14-20]. Adopting this precedent assured comparability. In addition, clinicians and clinical researchers often use "14 or more days" as a marker for clinical depression and anxiety disorders, and longer symptomatic durations are associated with higher levels of activity limitation [12,21]. Detailed definitions of the nine indicators are available in our previous paper [6] or are accessible via CDC's HRQOL website [12].

### Covariates

The authors examined twelve characteristics as potential confounders in the analyses. These included: five standard demographic measures (age, sex, race/Hispanic ethnicity, income, and employment); four health conditions (asthma, diabetes, obesity, and physical disability); and three health risk behaviors (smoking, chronic alcohol use, and no leisure physical activity). Current asthma status was ascertained by asking respondents, "Has a doctor ever told you that you had asthma", and then "do you still have asthma?" Diabetes status was ascertained by asking respondents, "Have you ever been told by a doctor that you have diabetes?" Responses were coded as "yes", "yes during pregnancy", or "no". Gestational diabetes was coded as "no" diabetes. Disability status was based on responses to two questions: "Are you limited in any way in any activities because of physical problems?" "Do you now have any health problem that requires you to use special equipment, such as a cane, a wheelchair, a special bed, or a special telephone?" Responses were coded as "yes" if they answered "yes" to either of these two questions.

Body mass index was calculated as weight in kilograms divided by the square of height in meters. A respondent was considered obese if their body mass index was ≥ 30 kg/m^2^. A current smoker was defined as someone who had smoked at least 100 cigarettes in their lifetime and who indicated they presently smoke every day or some days. Men were considered chronic drinkers if they drank an average of 2 drinks or more every day during the past 30 days, while women were considered chronic drinkers if they drank an average of 1 or more drinks per day during the past 30 days. A respondent was considered to be physically inactive if they did not participate in any leisure time physical activity or exercise during the previous 30 days [13]. Selection of these variables paralleled the methods employed by other studies which have examined relationships between a specific HRQOL indicator and various predictors [16,22], or which have examined multiple HRQOL indicators in relation to demographics [2,23], health risks [2,19,24], or specific health conditions [2,17,18,25-27]. In addition, our preliminary modelling identified these as important variables to retain while a number of others were eliminated. We dichotomized some variables for the analysis (i.e., sex, current smoking, alcohol use, physical activity, asthma, diabetes, obesity, disability), while others had multiple categories (i.e., age, race/Hispanic ethnicity, income, and employment status). The definitions of the 12 variables are available in our previous paper [6]. Reference groups chosen for the LCR model were those having the lowest risk for poor/fair general health and usually the lowest risk for the other HRQOL variables as well.

### Statistical analysis

The latent class regression (LCR) model was proposed initially by Dayton and Macready [28,29]. It aims to identify a set of classes of a latent variable from a set of observed discrete variables [30-32]. It also provides the probability of a particular individual belonging to a latent class [33]. The LCR model is a model for multiple indicators of latent classes. In contrast to logistic or linear regression models, it focuses attention on the set of latent classes identified in the analysis, rather than considering each of the observed indicators separately or all possible combinations of the observed indicators [30]. Detailed descriptions of the LCR model are available elsewhere [28,29,34,35].

In the LCR model, the unit of analysis is the response pattern [7,36]. A response pattern is the set of responses given by an individual to a set of indicator questions. In our study, there are nine indicators of HRQOL with a total of 512 possible response patterns (2^9^). The authors used the LCR model to group these 512 patterns into a much smaller number of classes.

The LCR model is specified in two parts: (1) a model for the relationship between the latent classes and the observed indicators; (2) a regression model for the relationship between covariates and latent class membership [34].

The LCR model has two fundamental quantities: the marginal and the conditional probabilities [30]. The marginal probabilities can be interpreted as the prevalence of each latent class, and they must sum to 1.00, indicating that in addition to being mutually exclusive, the classes are exhaustive. The marginal probabilities tell us what proportion of the population is located in each class. The conditional probabilities are the class-specific response probabilities of each indicator variable. The conditional probabilities are considered before considering the marginal probabilities of the classes [30,37].

The one-class model was fit first, followed by sequentially increasing the number of latent classes in order to determine the most parsimonious model providing an adequate fit to the data [31,33,36]. Having compared all the models, the LCR model with the optimal number of latent classes was selected. It is common in latent class analysis to fit models with different numbers of classes and compare them by Bayesian information criterion (BIC) and choose the model with the smallest BIC values [37,38]. Then, the prevalence of participants in each of the latent classes, and the conditional probability of the indicator variables for a participant in a given class, are assessed. Finally, the LCR model also makes it possible to estimate the effects of covariates on predicting latent class membership [39].

The study utilized the Mplus (version 3.11) software to implement these procedures, because it can accommodate the BRFSS weight variable. All models were estimated using maximum likelihood estimation. Ten sets of random starting values were specified for the final stage of maximum likelihood optimization to avoid the issue of local maxima and to ensure all values converge to identical solutions [32,33,37]. We obtained parameter estimates and standard errors of estimates for each indicator of poor HRQOL, in relation to each of the 12 independent variables. The t-test was used to identify statistically significant relationships (p (two-sided) ≤ 0.05).

In order to maintain maximal sample size and retain all valid data for the LCR, we simulated missing data for all variables using multiple imputation (MI). MI has been extensively applied to handle missing data in survey samples [40,41]. A basic assumption of MI is that missing data are missing at random [40]. In our study, 6 complete datasets were created by replacing missing values with simulated values. A detailed description of MI is available in our previous paper [6].

## Results

### Descriptive information

Frequencies and percentages for demographic characteristics, health risks, health conditions, and HRQOL indicators appear in Table 1. Overall, 14.85% had fair or poor general health. Results for the other 8 indicators based on the criterion of 14 or more days of poor health in the past month were as follows: 6.75% had activity limitations due to a physical or mental health problem; 10.55% had poor physical health; 9.7% had pain related activity limitations; 28.8% reported lack of energy; 23.8% reported inadequate sleep or rest; 13.2% were worried, tense or anxious; 10.5% had poor mental health; and 8.2% were sad, blue or depressed.

**Table 1:** Percentage for selected demographics, risk factors, health conditions, and HRQOL indicators among Rhode Island adults, 2004^†^

Demographics, risk factors & health conditions		n	%	HRQOL indicators	n	%
Age group	18–44 years	1524	51.0	Poor/fair general health	670	14.8
	45–64 years	1588	30.4	Activity limitation^‡^	311	6.7
	65 + years	837	18.5	Physically unhealthy^‡^	495	10.5
Gender	Men	1531	47.2	Pain related activity limitation^‡^	417	9.7
	Women	2468	52.8	Lack of energy^‡^	1117	28.8
Race/ethnicity	White, non-Hispanic	3367	84.7	Mentally unhealthy^‡^	455	10.5
	Hispanic	332	8.8	Sad/blue/depressed^‡^	343	8.2
	Other	244	6.5	Worried/tense/anxious^‡^	516	13.2
Income	< $25 k	960	24.9	Lack of rest/sleep^‡^	879	23.8
	$25 k-49,999	986	28.2			
	$50 k +	1519	46.9			
Employment	Unable to work	246	4.7			
	Unemployed	237	6.0			
	Homemaker/Student	298	10.3			
	Retired	795	17.3			
	Employed	2410	61.7			
Current smoker	Current smoker	820	21.3			
	Not current smoker	3168	78.7			
Chronic drinker	Chronic drinker	270	7.6			
	Not chronic drinker	3700	92.4			
Activity	Leisure time activity	1026	24.2			
	No leisure time activity	2971	75.8			
Asthma	Asthma	421	9.6			
	No asthma	3559	90.4			
Diabetes	Diabetes	328	7.2			
	No diabetes	3670	92.8			
Obesity	Obese (BMI>30)	762	19.0			
	Not obese	3016	81.0			
Disability	Have disability	717	15.3			
	No disability	3064	84.7			

†: Sample size is 3999.

‡: Criteria is >=14 days/month, see methods for complete variable description.

### Patterns of HRQOL

During the first stage of analysis, conventional latent class models, ignoring covariates, were fit to the HRQOL indicator data, demographics and health risks, starting with a 1-class model, and progressing to a model with four classes of HRQOL. The analysis indicated that the four-class model is the better model. During the second stage of analysis, when covariates were included in the models, the four-class LCR model with 12 covariates was selected as it had the lowest BIC score. The four latent classes are characterized as follows: Class 1 is characterized by physically and mentally good HRQOL; Class 2 was characterized as having physically related poor HRQOL; Class 3 was characterized as having mentally related poor HRQOL; and Class 4 as having both physically and mentally related poor HRQOL.

Table 2 presents estimates of (1) the marginal probability (proportion) of each of the 4 latent classes and (2) the conditional probabilities of each indicator for each latent class. RI adults in latent Class 1 (referred to as "healthy people"), accounted for 76% of the population; latent Class 2 (referred to as "physically unhealthy people"), comprised 9%; latent Class 3 ("mentally unhealthy people"), comprised 11%; and latent Class 4 ("both mentally and physically unhealthy people"), comprised 4% (see Table 2).

**Table 2:** Estimated parameters for the 4-class model

Indicators	Healthy people (Class 1)	Physically unhealthy people (Class 2)	Mentally unhealthy people (Class 3)	Both physically and mentally unhealthy people (Class 4)
Marginal probability (Proportion)	0.759	0.090	0.108	0.042
Conditional probability				
Poor/fair general health	0.062	0.682	0.081	0.749
Activity limitation^†^	0.000	0.383	0.054	0.658
Physically unhealthy^†^	0.017	0.641	0.061	0.702
Pain related activity limitation^†^	0.013	0.515	0.130	0.631
Lack of energy^†^	0.166	0.703	0.588	0.890
Lack of rest/sleep^†^	0.162	0.247	0.615	0.724
Worried/tense/anxious^†^	0.021	0.134	0.605	0.889
Mentally unhealthy^†^	0.017	0.122	0.430	0.866
Sad/blue/depressed^†^	0.002	0.037	0.336	0.936

†: Criteria is >=14 days/month, see methods for complete variable description.

Healthy people (class 1) have low probabilities (less than 17%) for each of the indicators of poor HRQOL. Conversely, both physically and mentally unhealthy people (class 4) have large probabilities (larger than 63%) for each of the poor HRQOL indicators. Physically unhealthy people (class 2) have high probabilities for the physical health indicators and low probabilities for the mental health indicators, while mentally unhealthy people (class 3) have low probabilities for the physical health indicators and high probabilities for the mental health indicators (see Table 2).

**Figure 1:**
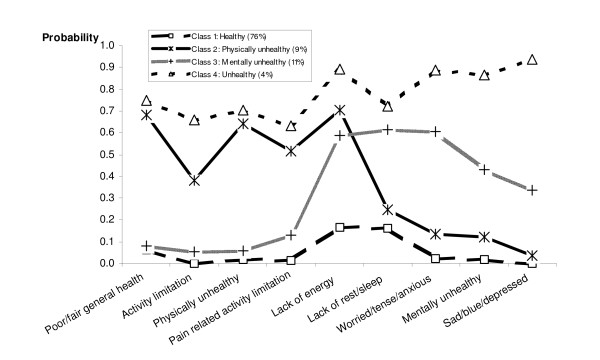
Latent class membership of Rhode Island adults in relation to HRQOL indicators.

Figure 1 is a diagrammatic representation of RI adults in latent classes 1–4. It visually demonstrates the unique divergence between Classes 2 and 3 and the magnitude of the difference between Classes 1 and 4.

### Predictors regressed on classes of HRQOL

The LCR model was used to determine which variables are significant predictors of latent class membership, when adjusting for all other variables in the model. Odds ratios are presented in Table 3 with latent Class 1 (healthy people) treated as the reference group.

**Table 3:** Demographic characteristics and risk factors regressed on three classes of HRQOL^†^

Demographics, risk factors & health conditions		Physically unhealthy (Class 2)^‡^	Mentally unhealthy (Class 3)^‡^	Both physically and mentally unhealthy (Class 4)^‡^
Age group	18–44 years	Reference	Reference	Reference
	45–64 years	1.75(1.01–3.01)*	0.72(0.46–1.11)	1.12(0.59–2.13)
	65+ years	2.39(1.04–5.47)*	0.22(0.10–0.49)***	0.29(0.09–0.89)*
Gender	Men	Reference	Reference	Reference
	Women	1.57(1.05–2.34)*	1.63(1.11–2.41)*	2.15(1.25–3.71)**
Race/ethnicity	White, non-Hispanic	Reference	Reference	Reference
	Hispanic	1.05(0.45–2.46)	0.64(0.25–1.60)	2.07(0.81–5.27)
	Other	1.20(0.49–2.90)	0.89(0.44–1.77)	1.28(0.46–3.58)
Income	<$25 k	1.56(0.87–2.80)	1.90(0.94–3.83)	3.67(1.50–8.98)**
	$25 k-49,999	0.97(0.56–1.68)	1.53(0.94–2.49)	1.39(0.57–3.38)
	$50 k +	Reference	Reference	Reference
Employment	Unable to work	6.35(2.98–13.54)***	0.80(0.17–3.78)	12.34(4.45–34.27)***
	Unemployed	2.25(1.05–4.84)*	1.67(0.79–3.51)	4.70(1.63–13.52)**
	Retired	0.96(0.50–1.86)	0.35(0.14–0.88)*	2.45(0.86–7.00)
	Homemaker/student	0.42(0.17–1.04)	0.72(0.32–1.62)	0.91(0.30–2.81)
	Employed	Reference	Reference	Reference
Current smoker	Current smoker	1.19(0.73–1.97)	1.93(1.26–2.95)**	3.26(1.90–5.61)***
	Not current smoker	Reference	Reference	Reference
Chronic drinker	Chronic drinker	0.96(0.32–2.92)	1.55(0.84–2.86)	0.74(0.10–5.35)
	Not chronic drinker	Reference	Reference	Reference
Activity	Leisure time activity	Reference	Reference	Reference
	No leisure time activity	3.04(1.98–4.67)***	1.20(0.68–2.12)	4.22(2.19–8.12)***
Asthma	Asthma	2.44(1.29–4.59)**	1.94(1.12–3.36)*	4.99(2.46–10.15)***
	No asthma	Reference	Reference	Reference
Diabetes	Diabetes	3.00(1.71–5.25)***	0.82(0.37–1.81)	3.68(1.75–7.74)***
	No diabetes	Reference	Reference	Reference
Obesity	Obese (BMI>30)	1.19(0.78–1.82)	1.54(0.97–2.45)	1.31(0.76–2.25)
	Not obese	Reference	Reference	Reference
Disability	Have disability	21.43(13.87–33.12)***	3.34(1.93–5.77)***	19.16(9.98–36.81)***
	No disability	Reference	Reference	Reference

†: Data are reported as adjusted odd ratios (AORs) by all other variables in the model, 95% confidence intervals (CIs) are reported in parentheses.

‡: Healthy people (Class 1) as reference group.

*: Statistically significant, ***p < 0.001; **p < 0.01; *p < 0.05.

Statistically significant results occur for each of the three latent classes for sex, current asthma, and disability. In general, women, people with asthma, and people with disability have greater odds of poor HRQOL in each Class than men, people without asthma, and non-disabled people. Disability is a highly relevant health condition for poor HRQOL. People with disability have exceptionally high odds ratios for each class of poor HRQOL, e.g. Class 2 OR = 21.43, Class 3 OR = 3.34, Class 4 OR = 19.16.

Being unable to work, unemployed, having no leisure time physical activity, and having diabetes were associated significantly with Classes 2 and 4. They predicted poor physical HRQOL among RI adults.

Current smokers were 1.93 times more likely to be mentally unhealthy than non-smokers, and 3.26 times more likely to be both physically and mentally unhealthy.

Table 3 shows that older age has a significantly increased association with membership in Class 2 (OR = 1.75 for 45–64 years and OR = 2.39 for 65+ years). On the other hand, increased age is related inversely to membership in Class 3 (OR = 0.72 for 45–64 year and OR = 0.22 for 65+ years).

The lowest income category was associated significantly with Class 4 (being both physically and mentally unhealthy) (OR = 3.67). There were no significant relationships observed for race/ethnicity, chronic drinking, or obesity.

To summarize: Class 2 was associated significantly with older age, being female, unable to work, disabled, or unemployed, having no leisure time physical activity, or having asthma or diabetes. Class 3 was associated with being female, being disabled, current smoking, or having asthma. Class 4 combined almost all the predictors of both Classes 2 and 3, e.g. being female, unable to work, disabled or unemployed, current smoking, having no leisure time physical activity, having asthma or diabetes, or having very low household income.

## Discussion

Several observations were made in utilizing a two-stage LCR model to determine latent classes of HRQOL. First, latent class models with different numbers of classes (1,2,3, and 4) were estimated initially without any covariates. Then, covariates (e.g., age, gender, race/ethnicity, income, employment, etc.) were included in the models. Since the inclusion of covariates changed the number of cases in each class, we found it was necessary to conduct classification simultaneously with class membership predictions [42]. Second, the local maximum is often encountered in likelihood estimation with LCR models. Thus, we used multiple sets of different starting values as recommended by [33,37]. Third, in choosing a LCR classification model it is important that each class have a reasonable number of observations, and that the latent classes estimated be interpretable [37].

As a result of our analysis, we have divided the Rhode Island population into four latent classes of HRQOL. The single class having good HRQOL has been labelled "healthy". The three classes having poor HRQOL have been labelled "physically unhealthy", "mentally unhealthy", and "both physically and mentally unhealthy". Three-fourths (76%) of Rhode Island adults are in the "healthy" class, while about one-fourth is in the "unhealthy" classes. The classes of "physically unhealthy" (9%), and "mentally unhealthy" (11%), together comprise 20% of RI adults, while 4% of adults are classed as "both physically and mentally unhealthy".

After controlling for all variables in the models, we identified the demographic characteristics, health conditions, and health risks having significantly increased odds, independent of one another, of being associated with one or more or the three classes of poor HRQOL. Other investigators assessing one or more of the HRQOL indicators in relation to demographics, health risks and health conditions have found similar associations [2,3,10,13,15,17,20,22,25].

### Disability, Asthma, and Gender

Significantly increased odd ratios for each of the three classes of poor HRQOL are associated with being disabled, having asthma, or being female in our study of Rhode Island's 2004 BRFSS data. The odds ratios for "physically unhealthy" and "physically and mentally unhealthy" poor HRQOL associated with being disabled were exceptionally high (OR = 21.43 and 19.16 respectively), and conform with findings of Strine et al. [13] who used the BRFSS to examine disability in relation to the individual indicators of poor HRQOL. Likewise, Strine [20], and Ford et al. [2] found that persons with asthma were significantly more likely than those without asthma to be at increased risk for several of the single indicators of poor HRQOL. Another study [43] found that people with asthma from Los Angeles county, CA experienced worse quality of life than people without asthma. These studies have also identified that women are significantly more likely than men to have poor HRQOL [13,20,44]. Women in the reproductive age group, who tend to carry more of the load than men for household labor, child-care, and parental care, frequently experience a substantial amount of physical and mental distress, depression, and stress or anxiety, and a high proportion of these women do not get enough rest or sleep [15].

Several subpopulations in our study had significantly increased odds ratios of having "physically unhealthy" as well as "physically and mentally unhealthy" poor HRQOL. These included: those unable to work, unemployed, lacking any leisure physical activity, or with diabetes. Other studies have also identified these same risk groups at high risk for poor HRQOL [3,43].

### Employment

Brown et al. [2] showed, after multivariable adjustment, that unemployed adults were twice as likely as employed adults to have poor quality of life. Unemployment may affect health directly; it can also provoke adverse risk behaviors, like smoking and heavy drinking [2]. Unemployed persons represent a population in need of public health intervention to reduce the burden of physical and mental distress.

### Physical inactivity

Unger [45] reported that the lack of any leisure physical activity was associated with a high risk of reporting poor physical health for men, and these relationships were significant only in the older age groups for women. Brown et al. [2] found an association between no leisure physical activity and HRQOL for both physical and mental health, but being physically unhealthy appeared to be more strongly associated with inactivity than being mentally unhealthy. Considering that one of the ultimate goals of Healthy People 2010 is to improve quality of life, these results highlight the need for health promotion programs that encourage physically active lifestyles and increase participation in regular physical activity [2,45].

### Diabetes

In a study among adults 50 years and older by Brown et al. [3,46], diabetes was associated with impaired physical health but not with impaired mental health, after multivariable adjustment. Preventing diabetes and its complications through health education that stresses a balanced diet and increased activity should be a public health priority [3].

### Smoking

Current smokers had increased odd ratios for "mentally unhealthy" and for "mentally and physically unhealthy" poor HRQOL. These findings of poor HRQOL are consistent with previous studies [6,47,48]. Lasser et al. [47] suggested that people with poor mental health are more likely to smoke than those who have good mental health. Strine et al. [49] found there is a significant association between smoking and impaired mental health, and current smokers were more likely to drink heavily, and to report mental health symptoms. Providing mental health services in conjunction with smoking-cessation programs, and vice versa, is indicated.

### Income

Having an annual household income under $25,000 in our study increased the odds ratios of having "mentally and physically unhealthy" poor HRQOL (OR 3.67), compared with the high income group. A strong relationship between low income and poor physical and mental HRQOL is consistent with the results of other research [6,46,50]. Ôunpuu et al. [48] showed low income is associated with health impairment. Kahn et al. [50] found women with young children in the lowest fifth of distribution of household income were at substantially higher risk of poor health and depression. Household income influences physical and mental health, which indicates the need to target interventions on such households.

### Older age

Our research found that the odds ratios of having "physically unhealthy" poor HRQOL was elevated for those ages 45 and older, and especially for those over age 65, compared with younger adults. CDC (Zahran et al.) [51] reported that low-income adults aged 45–64 years have worse HRQOL than all other adults. Unemployment, inability to work, and activity limitation partially explain these HRQOL disparities in this age-income group [51]. Targeting these risk factors and improving social services (e.g., job training programs) could help increase the quality and years of healthy life and eliminate health disparities for persons in this age group [51]. However, independent of these other covariates, our findings demonstrate the positive relationship between older age and physically unhealthy poor HRQOL, which is not surprising considering the vast array of physical ailments that are prevalent among older individuals. Public policy and interventions related to the promotion of healthier lifestyles and improved access and affordability of health care and medications should be targeted at this age group to improve and prolong physical health, longevity, and quality of life.

### Associations with better HRQOL

The odds ratios of having "mentally unhealthy" poor HRQOL in our study decreased with increasing age, and for retired people, compared with those currently employed. This observation likely reflects that older healthy and independent adults are more able to participate in the phone survey.

### Study limitations

There are five major limitations to our study and methodology. First, because of the cross-sectional design of the survey, we cannot determine the temporal relationship between classes of poor HRQOL and any of the risk factors. Future longitudinal studies are needed to investigate these relationships appropriately. Second, the BRFSS excludes households without land-line telephones, and adults living in institutional settings, such as group homes and nursing homes. Such exclusions undoubtedly underestimate the proportion of the adult population with compromised quality of life [46]. Third, self-reported data are affected by recall bias, that is over- and under-reporting of behaviors and existing disease [52]. Fourth, no statistical software package currently available for complex design survey data (e.g. SAS, SUDAAN, SPSS, STATA) can do any modeling other than logistic and regression analyses. We use a logistic regression analysis to test the difference between results obtained using survey design SAS procedures (SURVEYLOGISTIC) and standard SAS procedures (LOGISTIC). These two procedures are almost the same except that the SURVEYLOGISTIC procedure includes strata and cluster statements, while the LOGISTIC procedure does not include strata and cluster statements. The LOGISTIC Procedure uses the weight variable rescaled to sample size (wt = n*_finalwt/Σ_finalwt). The parameter estimates from the two procedures are the same. The standard errors are slightly different; the standard error from the LOGISTIC procedure is less than the standard error from the SURVEYLOGISTIC procedure, because the latter uses a sandwich-type robust estimator to account for strata and the sampling proportion. The sampling proportion is used to adjust the finite population. In addition, every individual in the BRFSS is a PSU, so the results with the cluster statement are the same as the results without the cluster statement. Whether we used _finalwt or the rescaled weight variable to run the SURVEYLOGISTIC procedure, the results are the same. When we do two Latent Class Regression analyses, one using _finalwt and one using the rescaled weight variable, the results are the same. Considering the acceptable difference between standard error estimates using LOGISTIC and SURVEYLOGISTIC, we have generalized this finding to LCR, which can be run with Mplus statistical software. Mplus can accommodate sample weights in performing latent class analyses, but not the strata used in the sampling design. Fifth, the limitations of the BRFSS survey meant some chronic health conditions which likely have a significant impact on quality of life, such as heart disease or cancer, could not be included in identifying high risk subgroups.

### Study advantages

Despite these limitations, the BRFSS is the only data source available for assessment of HRQOL in Rhode Island's adult population. Because it is a continuing annual survey, it allows us to track the prevalence of health behaviours, health conditions, and HRQOL over time and among populations at risk. Public health practitioners can use these data to target resources and interventions for both the mental and physical needs of subpopulations of Rhode Islanders with risk factors that are most associated with different types of poor HRQOL. Because it is easier to communicate about four classes of HRQOL than about each of nine individual HRQOL indicators, these classes provide an effective means of assessing progress towards RI's Healthy People 2010 goal of increasing the quality of life for Rhode Islanders [1].

## Conclusion

Using a LCR model we found four distinct classes of HRQOL among Rhode Island adults and were able to quantify the prevalence of each. The largest class has good HRQOL; three smaller classes have poor HRQOL, including physically unhealthy, mentally unhealthy, and both physically and mentally unhealthy. We also identified the demographic, health risk, and health condition characteristics of groups at high risk for the three classes with poor HRQOL.

The difference between our approach and others which have assessed the individual HRQOL indicators are: (1) we have created a meaningful and simple model to characterize and quantify population HRQOL; (2) we have identified subgroups within the population that have an elevated risk for three, two or one classes of poor HRQOL, these subgroups being: persons with disabilities, with asthma, with diabetes, who are unemployed or unable to work, women, smokers, and the elderly. These subgroups are identifiable and potentially reachable with creative policy and intervention initiatives. Focusing interventions on high-risk groups may be more beneficial in reducing the burden of poor physical and mental health and improving HRQOL for Rhode Island as a whole than if broad efforts are directed to the entire population. Furthermore, this strategy could certainly be more cost effective and could reduce the total economic cost of health care in the state.

Further investigation would be needed to gain a better understanding about the relationship between specific disease conditions, health risks, or demographics and compromised quality of life. Our results substantiate the need for ongoing support for individuals with specific chronic disease conditions (e.g. diabetes and asthma) to enhance their quality of life, and indicate how important early intervention and prevention are for these conditions. Our study also substantiates the importance of physical activity as a behavioral mediator that affects both health conditions and quality of life. Furthermore, it calls particular attention to the critical importance of having adequate mental health services if quality of life is to improve for Rhode Island's population.

## List of abbreviations

BRFSS: Behavioral Risk Factor Surveillance System; CDC: Centers for Disease Control and Prevention; CI: Confidence Interval; HRQOL: Health-Related Quality of Life; LCR: Latent Class Regression Model; MI: Multiple Imputation.

## Competing interests

The authors declare that they have no competing interests.

## Authors' contributions

YJ contributed to the preparation of the database, conducted the literature review, collaborated on analytic decisions and data interpretation, performed the statistical analyses, prepared the data tables, and drafted the manuscript. JEH manages the RI BRFSS, collaborated on analytic decisions and data interpretation, and revised and edited the manuscript. Both authors have read and approved the final version of the manuscript.
